# Ecuadorian electrical system: Current status, renewable energy and projections

**DOI:** 10.1016/j.heliyon.2023.e16010

**Published:** 2023-05-01

**Authors:** Daniel Icaza-Alvarez, Francisco Jurado, Carlos Flores, Geovanny Reivan Ortiz

**Affiliations:** a Department of Electrical Engineering, University of Jaen Linares, Spain; b Department of Electrical Engineering, Catholic University of Cuenca, Cuenca, Ecuador

**Keywords:** Renewable energy, Energy policy, Energy transition, Energy law, Concession, Reform to the law

## Abstract

In this research, an analysis of the electricity market in Ecuador is carried out, a portfolio of projects by source is presented, which are structured in maps with a view to an energy transition according to the official data provided. State policies are analyzed, as well as the opportunities for development in renewable energies offered by the reform of the Organic Law of the Electric Power Public Service. Additionally, the roadmap is presented, which includes an increase in the levels of renewable energies and a decrease in fossil fuels in consideration of the growing demand for electrical energy with a view to 2050 in accordance with the state approaches defined in recent years. It is considered that the total 100% renewable installed capacity by 2050 is 26,551.18 MW compared to 11,306.26 MW in 2020 between renewable and non-renewable. It is expected that the current legal framework will continue to articulate strategies for a greater penetration of renewable energies, that national purposes be achieved and international agreements celebrated both regionally and globally be fulfilled, so it is important that sufficient resources are allocated to achieve this long-awaited energy transition in Ecuador.

## 1 Introduction

Electric energy is vital for the economic development of countries and the improvement of people's well-being. Electric energy is what mainly makes factories and businesses work and allows us to enjoy a comfortable environment in our homes. For this reason, all the countries where the economy is growing also register an increase in their energy consumption, which also includes aspects such as mobility, heating and cooling [1]. The energy area becomes a key strategic concern for governments, especially if it is considered a public service for which the state is the guarantor of providing the service to all citizens [2]. Studying the energy systems in a region or country implies understanding its history and in what proportion it evolved, the implications of the reforms to the law and the possibilities of modernization, especially with the support of renewable energies. In Ecuador, as of 2015, the reforms to the law of the Electricity sector were concretized that allowed a substantial development compared to previous decades, it is the so-called “Organic Law of the Public Electricity Service (LOSPEE)" [3]. The advantage with respect to the previous law, this reform allowed increasing government participation with greater investments in infrastructure with a legal framework in accordance with the Constitution achieved in Montecristi in 2008 for the Republic of Ecuador [4]. In this way, the modernization of the electricity sector began to be encouraged to provide end users not only with a public service of high-quality electricity, but also with reliability and safety [5]. It is important to indicate that the Ecuadorian State is the one who supervises the management of the companies as well as their profits at the level of generation, transmission and distribution [6].

However, in order to stimulate investment, the State is also in charge of developing certain logistics projects considered strategic, which aim to create the conditions for investors to carry out their activities with all possible guarantees [7]. Thus, directly or in partnership with private capital, the State is in charge of building, among others, power generation plants, an essential public work so that production can circulate to consumer markets, whether national or international [8]. The Administration (understood as the State) intervenes in this way indirectly in the economy by enabling the necessary infrastructure to facilitate the production or trade of goods, thus it is concerned with contributing to the interest of economic agents by creating the logistics of support for their activities, and also indirectly promotes or stimulates production and marketing [9].

Among the main findings that this research presents after making projections to 2050, it is recommended that hydraulic energy reach 8500 MW from 2030 and remain at this value until 2050, including the portfolio of currently planned projects. On this basis, new wind and solar photovoltaic technologies are included with important proportions of 8890 MW and 4000 MW respectively. Other renewable energies are also contemplated in a smaller proportion that should not be neglected for any reason and rather diversify the energy mix. This change in the energy matrix that is outlined in Ecuador agrees with the literature that is raised worldwide and is emphasized by researchers such as TL Afonso et al. [10] and B Doğan et al. [11]. Along the same lines, Southeast Europe [12] is already on the path to this transition process based on renewable energy. The road map designed in Japan [13] and in a select group of African countries [14] includes a considerable increase in wind and photovoltaic solar energy with the purpose of diversifying the respective electrical system. In this line, Ecuador advances in the right direction and in this article the route of energy development is traced, attached to the literature and experiences of other parts of the world.

The main objective of this article is to present the current state of the Ecuadorian electricity sector, make renewable energy projections based on renewable energy potential, future projects and the growing demand estimated by the MERNNR. The evaluation carried out will allow a better use of renewable energies to propose an orderly energy transition, defined in the State Laws, emphasizing the evolution, reforms and current structure for its growth and modernization. These aspects are necessary for a correct understanding of the problems of the Ecuadorian electrical system and its development paths in the long term, allowing to know its management and energy transition policies. The generation projections are carried out with the support of specialized software EnergyPLAN.

The document is organized as follows: Section 1 presents the introduction referring to the Ecuadorian electrical system, referring that it will be analyzed as a case study and the methodology. Section 2 presents the socioeconomic situation in Ecuador. Section 3 contains the projection of electricity demand by consumption sectors. Section 4 presents an analysis of the electricity sector for the use of renewable energies as an appropriate option for an energy transition. Section 5 presents the Reform to the law and its implications in the generation of renewable energy. In section 6 the case is analyzed and discussed and finally in section 7 it is present the conclusions.

### 1.1 Methodology

The methodology in this study assumes the existence of sufficient renewable energy resources for energy use and subsequently assesses the technological impacts. The three phases that involve the analysis of the strategy consist of: Data entry of the demand by sectors presented in Section 2 and the useable resources in the territory that is considered in Section 3, adjustment or regulation that is carried out using specialized software such as EnergyPLAN and the results are presented in Section 4. Finally, the results are evaluated and the scenario up to 2050 is presented. Indirect aspects are also evaluated, such as technological renewal in energy matters, in the economic aspect they include services at affordable prices, ensuring flexibility from the technical and economic point of view.

## 2 Socioeconomic aspect of Ecuador

Ecuador, if It is located in South America, has an approximate area of 256,370 km^2^ and a population of 17,888,474 people according to [15]. It is in position 67 of the population catalog, made up of 196 countries. It has a moderate population density, around 70 inhabitants per km2. The capital is Quito and it has two development pole cities such as Guayaquil and Cuenca, its currency is US dollars. Ecuador is the 65th economy by volume of GDP. Its public debt at the end of 2020 was 53,050 million euros, with a debt of 60.89% of GDP [16]. Its per capita debt is €3030 euros per inhabitant according to figures presented by (Ecuador, 2022.). The latest annual variation rate of the CPI published in Ecuador at the end of June 2022 was 4.2%. The main source of energy in Ecuador continues to be Petroleum. The abundance of this non-renewable resource has allowed the country to position itself as a net exporter of oil as the most prominent export product. The discovery of new oil fields in the country indicates a dangerous reliance on a single energy source to the point that some researchers recommend diversification. They highlight the initiative proposed by former President Rafael Correa to leave crude oil on the ground and thus avoid an ‘economic mirage’, from the beginning of the ‘oil boom’, between 1972 and 1982 [17]. Currently, President Guillermo Lazo issued Decree No.- 59 renaming the Ministry of Environment and Water to the Ministry of Environment, Water and Ecological Transition, with the intention of achieving sustainable development while respecting the rights of nature as it is proclaimed in the constitution [18].

The GDP per capita is an important indicator that evaluates the standard of living, in the case of Ecuador, in 2021 it was 5017 euros, which ranks 99, being a parameter that presents a very low level [19]. Regarding the Human Development Index (HDI), which shows the standard of living of its population, the country is in position 86. On the other hand, it is interesting to know that Ecuador is in position 123 of 190 according to the [20], that is, they present ease of doing business.

The Ecuadorian electricity sector is considered strategic due to its direct influence with the development productive of the country. In Ecuador for the year 2020, the generation capacity registered in the national territory was 8712.29 MW of NP (nominal power) and 8095.25 MW of PE (Effective power). The generation sources are presented in Table 1.

**Table 1:** Electrical generation sources in Ecuador [21].

Source	Central	Unit Type	Nominal Power (MW)	Effective Power (MW)
Non-Renewable	Thermal	MCI	2029.74	1633.25
		Turbogas	921.85	775.55
		Turbo steam	461.63	431.50
TOTAL Non-Renewable			3413.22	2840.30
Renewable	Biomass	Turbo steam	144.30	136.40
	Wind	Wind	21.15	21.15
	Hydraulics	Reservoir	1733.20	1749.60
		Pass	3365.55	3314.56
	Photovoltaic	Photovoltaic	27.63	26.74
	Biogas	MCI	7.26	6.50
TOTAL Renewable			5299.09	5254.95

The great investment and the multiple efforts carried out, both by government and private entities, have contributed to the achievement of an electrical matrix with a high participation of hydroelectric generation and a reduced thermoelectric generation and to the strengthening of the transmission, sub-transmission and distribution networks, adapting them to current and future electricity supply and demand conditions [22]. In this way, electricity demand is presented as an essential indicator for planning the sector.

Table 2 contains the historical evolution of the annual power demand in Ecuador (from 2010 to 2020), according to data officially provided by the Agency for the Regulation and Control of Non-Renewable Energy and Natural Resources.

**Table 2:** Annual electricity demand in Ecuador 2010–2020. Source [21]:

Año	2010	2011	2012	2013	2014	2015	2016	2017	2018	2019	2020
January	2720.83	2910.66	2939.16	3190.31	3324.28	3504.00	3593.10	3689.18	3815.28	3903.44	4083.08
February	2740.63	2932.09	3036.78	3151.74	3324.14	3523.27	3638.11	3645.86	3748.54	3906.90	4089.12
March	2819.60	2963.85	3014.22	3214.05	3369.52	3540.40	3654.22	3692.24	3905.45	3886.47	4032.18
April	2836.18	2951.51	3091.88	3234.29	3402.35	3606.74	3583.04	3683.19	3902.63	3941.81	3458.73
May	2834.44	2979.65	3088.18	3185.68	3396.90	3601.99	3586.75	3687.69	3816.81	3949.94	3626.89
June	2732.30	2877.66	3041.94	3107.99	3399.01	3559.68	3624.79	3561.15	3673.05	3778.59	3633.50
July	2695.20	2841.57	2990.20	3039.13	3352.43	3525.24	3450.27	3435.24	3617.14	3701.49	3650.21
August	2699.00	2831.19	2983.52	3080.53	3292.97	3471.17	3490.36	3577.25	3585.30	3668.14	3712.96
September	2742.00	2897.34	3058.91	3218.77	3307.95	3544.75	3490.36	3577.25	3799.52	3697.72	3820.26
Octuber	2879.04	2891.36	3035.26	3187.60	3373.11	3591.02	3457.48	3674.02	3657.19	3790.12	3935.11
November	2815.88	2999.81	3125.07	3277.04	3423.45	3653.34	3572.86	3586.63	3773.64	3953.33	3921.50
December	2879.24	3052.29	3206.73	3332.49	3502.64	3669.58	3624.67	3745.77	3856.97	3951.68	
**Maximum Power (MW)**	**2879.24**	**3052.29**	**3206.73**	**3332.49**	**3502.64**	**3669.58**	**3654.22**	**3745.77**	**3905.45**	**3953.33**	**4089.12**
**Minimum Power (MW)**	**2695.20**	**2831.19**	**2939.16**	**3039.13**	**3292.97**	**3471.17**	**3450.27**	**3435.24**	**3585.30**	**3668.14**	**3458.73**
**Average Power (MW)**	**2782.86**	**2927.42**	**3050.99**	**3184.97**	**3372.40**	**3565.93**	**3563.83**	**3629.62**	**3762.63**	**3844.14**	**3827.17**

On the other hand, Table 3 organizes the data corresponding to the power demands at generation terminals (MW) and energy (GWh), corresponding to the years 2019 and 2020, which follow the behavior illustrated in Fig. 3.

**Table 3:** Comparison between electricity demand at generation terminals (MW) and energy demand (GWh) in Ecuador between 2019 and 2020.

	Demand at generation terminals (MW)			Energy demand (GWh)		
	2019	2020	% Change	2019	2020	% Change
January	3903.44	4083.08	4.60	2096.56	2264.85	8.03
February	3906.90	4089.12	4.66	1946.52	2129.57	9.40
March	3886.47	4032.18	3.75	2150.00	2105.54	−2.07
April	3941.81	3458.73	−12.26	2117.26	1810.20	−14.50
May	3949.94	3626.89	−8.18	2162.90	1946.12	−10.02
June	3794.42	3633.50	−4.24	2000.16	1936.72	−3.17
July	3701.49	3650.21	−1.39	2042.18	1986.82	−2.71
August	3668.14	3712.96	1.22	2034.04	2002.61	−1.55
September	3697.72	3820.26	3.31	1974.23	2033.64	3.01
Octuber	3790.12	3935.11	3.83	2040.53	2166.47	6.17
November	3953.33	3921.50	−0.81	2059.80	2069.26	0.46
December	3951.68	3942.10	−0.24	2129.10	2183.90	2.58
**Total**	3953.33	4089.12	3.43	24753.23	24635.69	−0.47

Specifically, the maximum power demand was recorded on Thursday, February 06, 2020 at 7:30 p.m. and the maximum accumulated energy demand was in the month of February [22].

**Fig. 1:**
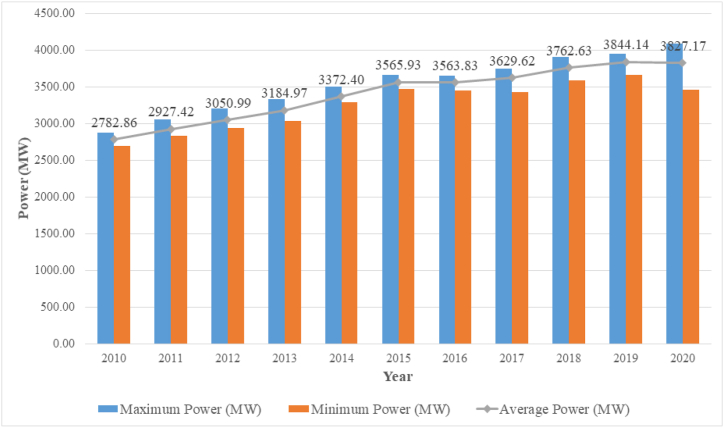
Historical electrical demand for maximum, minimum and average power 2010–2020. Source [21]:(adapted).

**Fig. 2:**
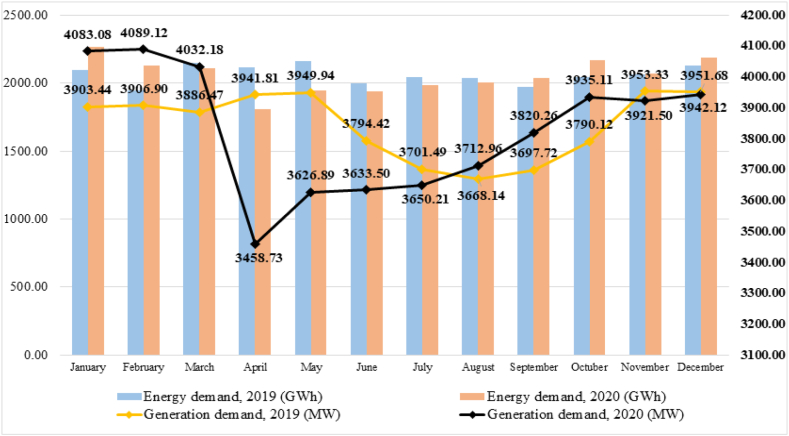
Power demands at generation terminals (MW) and energy (GWh), 2019 and 2020 [23].

In Fig. 1, Fig. 2, a marked decrease in electricity consumption can be seen after March 2020, coinciding with the beginning of the exception period decreed by the Ecuadorian state as a result of the global pandemic by COVID 19 (March 16, 2020), showing a minimum of 3458.73 MW in April (14.22% less than the previous month).

**Fig. 3:**
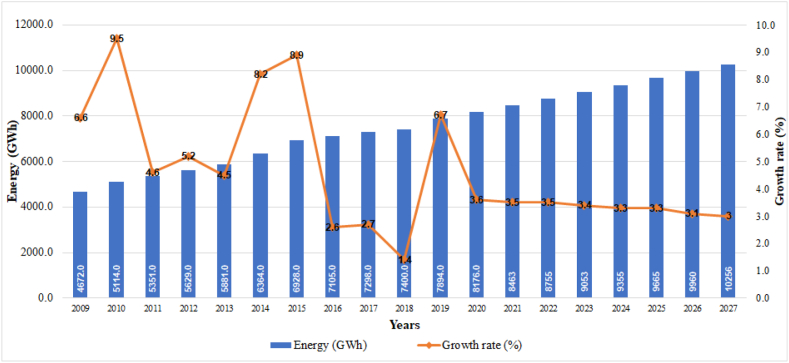
Historical evolution and projection of consumption in the residential sector.

Starting in April, the demand for power began to increase, reaching 3921.50 MW in November, which represents −0.81% of the value registered the previous year, that is, 31.83 MW less. This increase is attributed to the progressive reactivation of the different productive sectors of the country, considering that an important part of the labor market was adjusted to the remote work or telework modality, with which residential consumption is adjusted by increasing by 2%.

## 3 Official projection of electricity demand

The methodology used in the projection of Ecuador's electricity demand, considered variables of a technical, economic and demographic nature [24]; based on 4 large groups of consumption: residential, commercial, industrial, and public lighting.

### 3.1 Residential sector demand projection

The historical evolution of energy consumption in the residential sector during the period 2009–2020, and its projection until 2027, are illustrated in Fig. 3. As a data of interest, the effect produced by the demographic increase was not taken into account, given the reduced variability presented by the population in the last three intercensal periods. Only the effects that the future variability of GDP would produce are considered [24].

The projected consumption for the residential sector estimates an average increase of 3.7% between 2019 and 2027, for a total of 10,256 GWh at the end of the study period. This trend results mainly from vegetative growth and the beneficiaries of the rural electrification program; in this sense, the average consumption per residential user is expected to reach 1.81 MWh/year by 2027.

### 3.2 Commercial sector demand projection

**Fig. 4:**
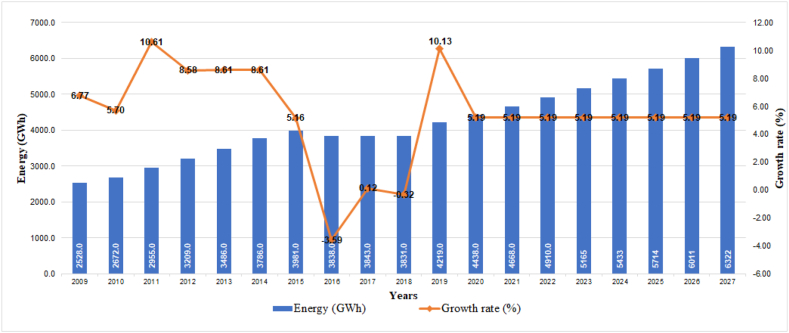
Historical evolution and projection of consumption in the commercial sector.

For the commercial consumption group (Fig. 4), the MERNNR forecasts an average subscriber growth rate of 3.27%, which corresponds to almost 650,000 users in 2027, who will demand around 6322 GWh (5.74%).

### 3.3 Industrial sector demand projection

**Fig. 5:**
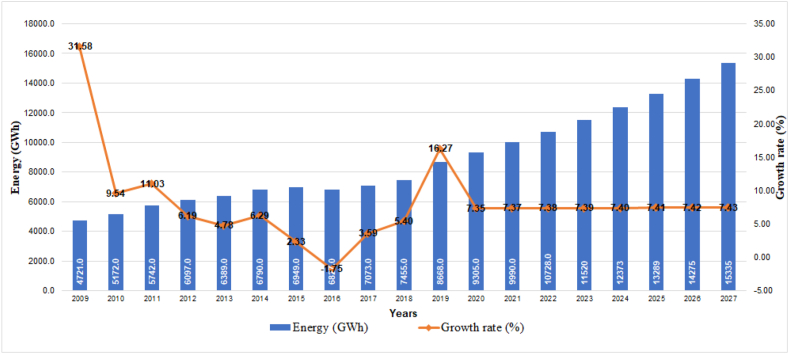
Historical evolution and projection of consumption in the industrial sector.

The development of the country has driven the growth of the industrial sector at an average annual rate of 3.03% (Fig. 5), a trend that will continue according to projections until 2027, when it is estimated to reach 160,619 users equivalent to 3.10% [24]. Said variation will trigger an 8.38% average annual growth for the year 2027, translated into a total of 15,335 GWh demanded.

### 3.4 Public lighting sector demand projection

**Fig. 6:**
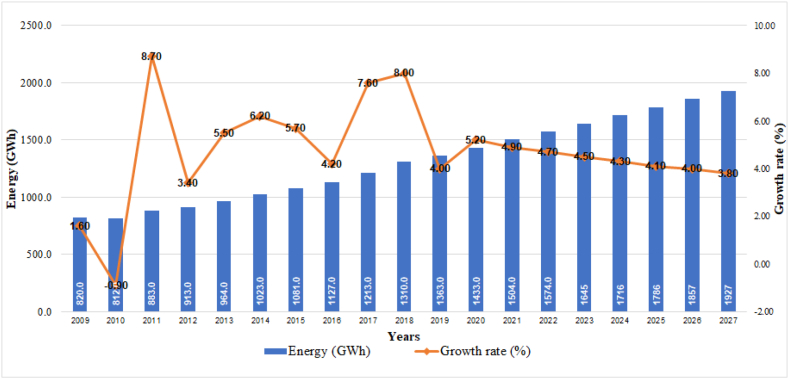
Historical evolution and projection of consumption in the public lighting sector.

According to data provided by Ref. [25], an average annual growth rate of 4.4% (1927 GWh) is expected by 2027, (Fig. 6).

### 3.5 Projection of electricity demand by sectors

**Fig. 7:**
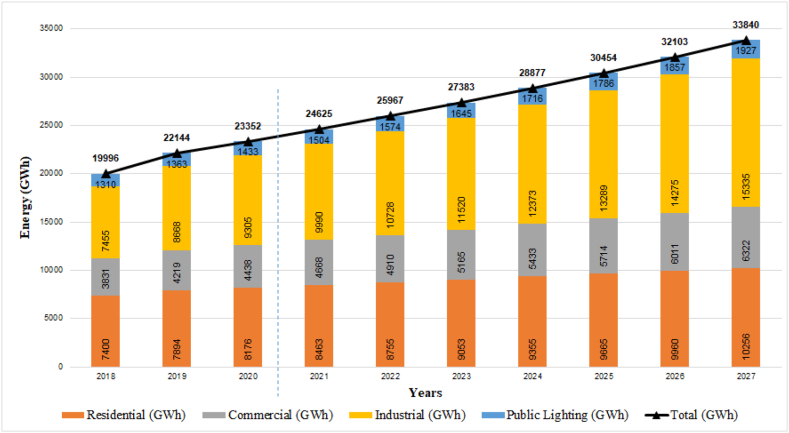
Projection of energy demand by consumption groups.

With an average annual growth rate of 2.43%, an approximate 6.48 million users are estimated in 2027, of which it is expected that: 87% belong to the residential sector, 10% to the commercial sector and the 2% to industrial, dismissing the participation of public lighting as marginal. Under such circumstances, the total energy demand will reach 33,840 GWh (5.44% annual average growth rate) in 2027, as indicated in Fig. 7.

The demand for energy projected by consumption groups must be fully satisfied, contemplating a greater generation of energy. Having the purpose of an energy transition entails increasing the levels of renewable energy generation and replacing fossil fuels. The task is greater, so it is essential that the projects that will be indicated in section 4 are the best directed and bring them to reality, with energy rates that do not considerably affect citizens.

## 4 Analysis for the use of clean energy

At the beginning of the pre-industrial era, GHG emissions had a value of 298 parts per million (ppm), later increasing to 398 ppm and 407.8 ppm in 2014 and 2018, respectively [26], pointing to a significant rise in recent years. Additionally, scientists have reported a 1 °C increase in global temperature, which should not exceed 1.5 °C until the end of the century, since the higher the temperature, the more extreme weather events increase in number, frequency and intensity. The only way to stop this is to achieve net zero emissions as soon as possible.

However, the planning of improvements to a power system must be carried out maintaining criteria of safety, reliability, efficiency, quality and continuity of service. Recalling Antonio Borrero, Manager of the Empresa Electro Generadora del Austro (ELECAUSTRO), that the intrinsic operational aspects of each energy source must always be taken into account [27], for example.

• The photovoltaic and wind power plants work under normal conditions for considerable values of solar irradiation (during the day) and wind speed, respectively.
• In times of drought, hydroelectric plants transfer part of their load to thermoelectric plants; hence the essential role of the latter as backup in case of contingencies.

According to the 2019 National Energy Balance report, 91.3% of the energy consumed in Ecuador came from fossil fuels [28], and 8.7% from clean sources, which translates into an increase of 4.2% in the use of this last type of resource in relation to the previous year. Despite these efforts, the country's energy production continues to show a high degree of dependence on fossil fuels, making it an urgent need to migrate to clean energies that have so far brought significant benefits to the country.

**Fig. 8:**
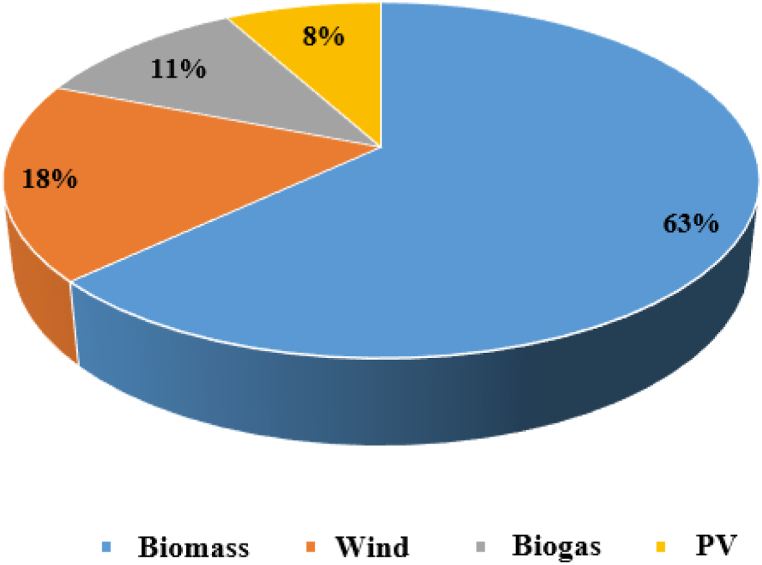
Gross energy production by type of generation (GWh), 2020 [29].

For the year 2020, Ecuador's energy production reached 27,120 GWh [23], which represents a reduction of 2.21% compared to the previous year; Seen from another perspective, 90.72% of the energy originated from clean sources; with an indisputable first place of hydroelectric participation (98.37%), and a percentage distribution of non-conventional energies illustrated in Fig. 8.

### 4.1 Interest to activate investments

Multiple transnational companies see Ecuador as an optimal place for the development of electrical projects associated with clean energy, thanks to: its hydraulic and solar potential, due to its geographical characteristics (location, relief, water resources, among others); its wind potential, in the Andes region; and, its biomass potential, derived from the high levels of residues derived from agricultural activity [30].

Ecuador has a Master Electricity Plan projected until 2027, which brings together a set of planned projects in order to cover the country's electricity demand, promoting private investment [31].

With this approach, the execution of the Non-Conventional Renewable Energy Block (NCREB I - 500 MW) is considered [32]. There are four unconventional technologies (hydroelectric, photovoltaic, wind and biomass) for projects located in different geographical areas of the country [33]. They will be located based on the primary resource, the environmental and logistical conditions of the site [34].

### 4.2 Location of renewable energy projects with a view to an energy transition

As part of the central government's policies, the MERNNR promotes and carries out Public Selection Processes (PPS) for concession to private capital companies, which among others are the power generation activities electricity in Ecuador. It should be noted that although these state portfolio projects have been defined, it does not mean that projects cannot be carried out in other sites with similar or relatively lower potential for solar energy generation [35]. The locations of the projects are located in different provinces, which is detailed in this same subsection.

#### 4.2.1 Hydraulic energy

**Fig. 9:**
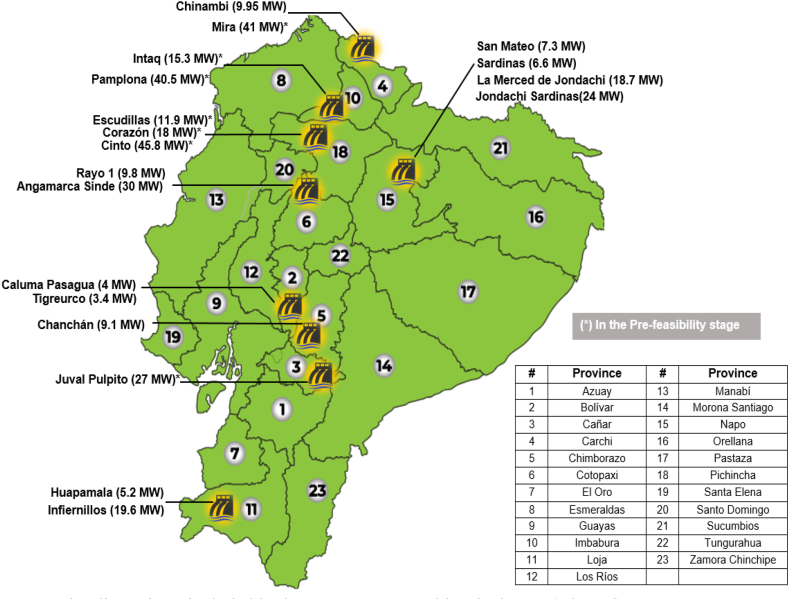
Hydraulic projects included in the 500 MW renewable Block [36] (adapted).

Previous studies concluded that the Amazon slope concentrates the greatest hydraulic potential in Ecuador (9.93 GW), the development of projects on the Pacific slope (3.5 GW) is pursued, preventing the imbalance of generation in rainy seasons [36]. The Electricity Master Plan contemplates the construction and entry into service of new hydroelectric plants in response to the growth in energy demand. Below is Fig. 9, which identifies the main hydroelectric projects in Ecuador.

#### 4.2.2 Solar energy

**Fig. 10:**
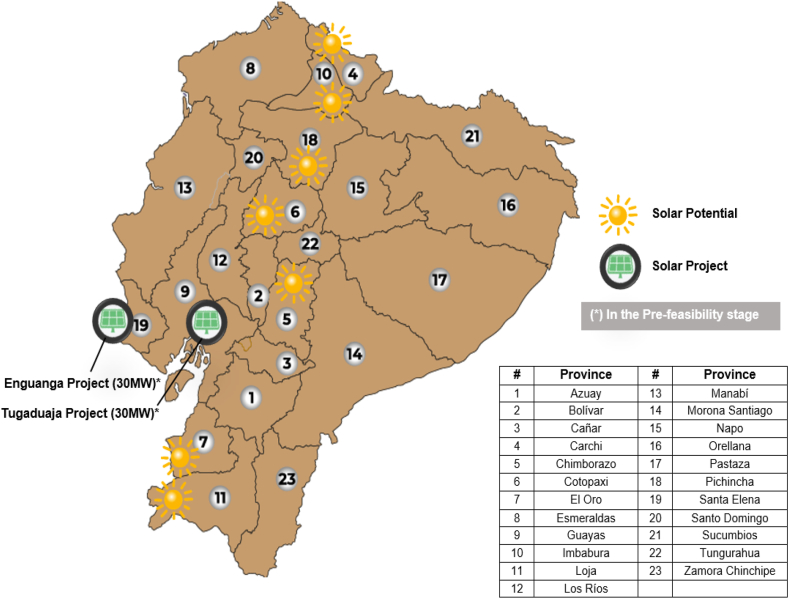
Solar projects included in the 500 MW renewable Block [36] (adapted).

The useable solar potential of the country, for its part, is estimated at 660 photovoltaic MWp, located in places with a high level of irradiation, feasibility of connection and areas that do not present environmental, social, etc. limitations, such as: Carchi, Pichincha, Cotopaxi, Manabí, Imbabura, Chimborazo, El Oro, Loja and Guayas (Fig. 10). Said potential lies in the levels of annual irradiance: maximum, 6.4 Wh/m2day; minimum, 2.8 Wh/m2day; and average, 4.5 Wh/m2day [37,38].

#### 4.2.3 Wind energy

**Fig. 11:**
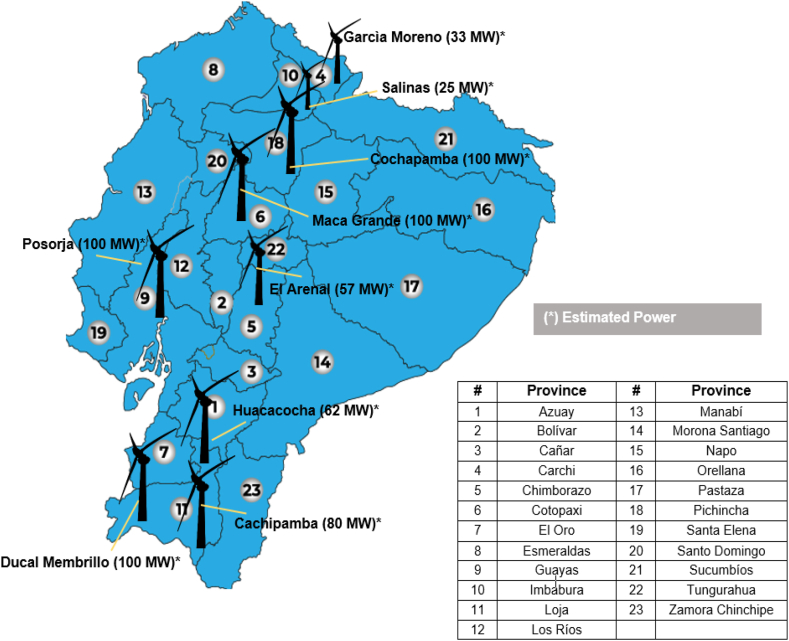
Wind projects for 25-year concession [36] (adapted).

According to the wind atlas of Ecuador [36,39], in the useable areas, the average annual wind speeds exceed 7 m/s at 3000 m above sea level, indicating a feasible potential of 891 MW in the short term, which would be added to the 21.15 MW of power in service (16.5 MW on the mainland, and 4.65 MW on the insular region). To date, measurements have been certified that prove the feasibility for the development of 9 continental implementation projects that would add 115 MW to the country's electrical system (Fig. 11).

#### 4.2.4 Biomass energy

Biomass in Ecuador is a resource that, for electricity generation purposes, comes mainly from the processing of sugar cane, African palm and rice husks [36] since this type of resource is available in almost all the regions of the country, its exploitation must consider the rapid organic decomposition, when planning the transport and storage of the raw material.

**Fig. 12:**
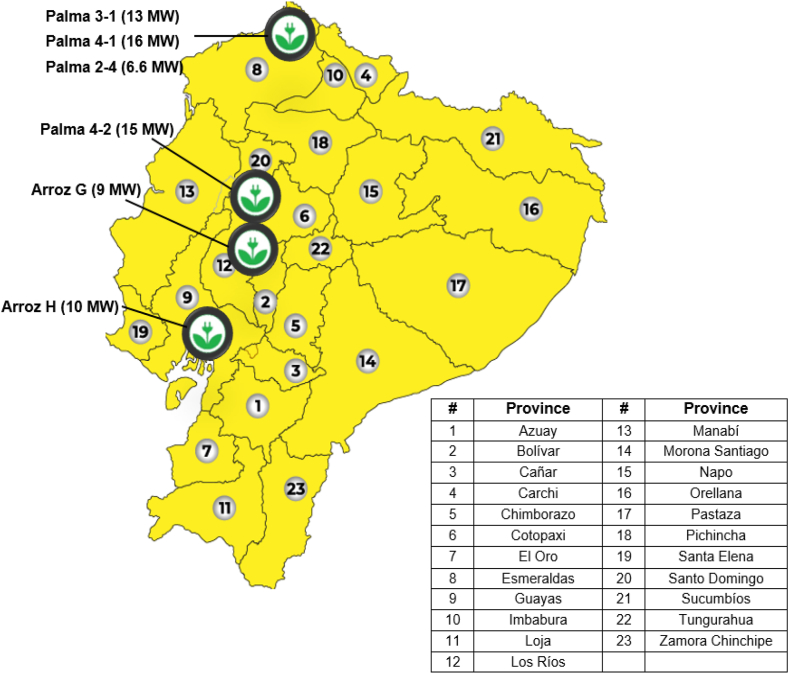
Projects for the use of biomass 2018–2027 [36] (adapted).

The Bioenergetic Atlas of Ecuador developed since 2015 [40], details the main characteristics for the use of biomass in the country's electricity generation; It considers 18.4 million tons per year of agricultural, livestock and forestry waste, from which approximately 12,700 GWh/year can be extracted. The projects contemplated in the Electricity Master Plan 2018–2027 [36,41], are illustrated in Fig. 12.

Based on what has been described, it is identified that there is a high potential for electricity generation in Ecuador, especially the types of projects and specific places to start them up by the central state and radicalize the energy transition. To this end, it will be necessary to “change the form of use of non-renewable energy, such as diesel, considering for now this first block of NCRE through hydroelectric, wind, photovoltaic and biomass, which have a clean generation matrix.

## 5 Reform to the law and its implications in the generation of renewable energy

The regulation called Organic Law of the Public Service of Electric Energy, (LOSPEE, 2015) promulgated on January 16, 2015, determines the management of energy sources and non-conventional renewable energies. Among its objectives is to guarantee the electricity service with the highest quality and reliability, encouraging modernization for the provision of the service.

With the enactment of the LOSPEE law, the electricity service has been substantially improved, especially the risk of cuts and rationing in the country no longer exists, however the goals set have not yet been fully met, mainly due to economic barriers, changes in government policies and confrontation between indigenous groups and the government over subsidies. Therefore, there is no regulatory policy that requires allocating substantial economic resources and allows achieving the LOSPEE purposes of implementing new systems, expanding and diversifying energy generation in the future to reduce the proportions of fossil fuels currently used.

### 5.1 Structure for the generation of non-conventional renewable energies

The current Ministry of Energy and Mines is promoting the use of alternative energies, in accordance with the provisions of the Constitution that proposes to develop a sustainable electrical system, mainly using renewable energy resources.

Electricity generated based on renewable resources includes preferential conditions and incentives established through regulations issued by ARCONEL.

### 5.2 Qualifying titles

The Ministry of Electricity and Renewable Energy, will be in charge of process and issue the following qualifying titles.

1. Operation authorization; and,
2. Concession contract.

In the case of mixed, private, economy companies popular and supportive, foreign state companies or subsidiaries of these, or consortiums in which said state-owned companies have a majority stake, the terms of duration of the qualifying titles are determined based on a financial analysis, which allows firstly, the amortization of the investments to be performed and obtain a reasonable profit; and, second, the importance of technical input, economic and social for national development.

In the case of self-generators, the term of the title enabling will be established considering the useful lives of the different types of technologies, excluding the principle of reasonable utility. The autogenerator decide to manage the obtaining of a qualifying title, should consider within the financial analysis, that the coverage of your investment and operating costs and maintenance, will be financed through the business of self-generation.

### 5.3 Operation authorization

The Ministry Electricity and Renewable Energy will process and issue the respective authorization of operation for the execution, operation and functioning of projects developed by public and mixed companies.

In the case of joint ventures, the authorization of operation will be considered as a delegation that grants the State, in accordance with article 316 of the Constitution of the Republic of Ecuador.

The requirements and procedures for the granting of the Operation Authorization after its approval, as well as the rights and obligations of the grantor and concessionaires, will be established in this law and its general regulations and the respective qualifying titles.

The Ministry of Electricity and Renewable Energy will sign contracts for concession with private companies and popular economy and solidarity, whose projects have been included in the PME or those that, by not appearing in the PME, have been proposed by the aforementioned companies, observing the regulations issued for this purpose.

The requirements and procedures for the granting of the concession contract after its approval, as well as the rights and obligations of the grantor and concessionaires, are established in the law and its regulations general application and the respective qualifying titles.

The promotion of non-conventional renewable energies, as well such as the use of renewable energy resources, must have prior authorization for the use of those resources by the Authority National Environmental Law, and must observe the provisions of the governing body of national planning.

### 5.4 Long-term transition process

**Fig. 13:**
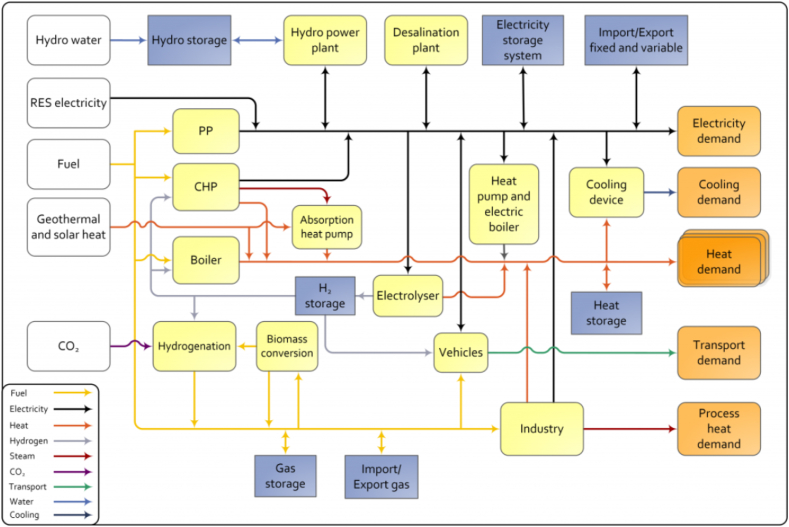
The energyPLAN model in version V16.0 [43].

Currently, several models allow long-term energy planning. In this work the concept of intelligent energy systems is assumed. The smart energy system detects and uses synergies between different sectors of the electrical system, that is, the general data provided in section 3 to make the respective projections. The EnergyPLAN model is developed and updated by Aalborg University in Denmark and is freely accessible [42], see Fig. 13.

In this research, EnergyPLAN is selected because it is a much more flexible tool to achieve the goal of reaching 100% renewable energy by 2050.

**Fig. 14:**
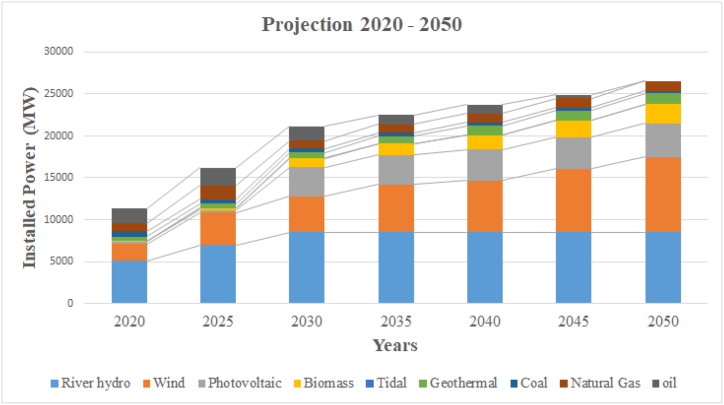
Proposal for the energy transition according to the proposed state projects and useable energy potential in Ecuador by 2050.

Table 4 and Fig. 14 present the proposed scenario to carry out the complete energy transition of the Ecuadorian electricity system derived from decree 59 issued by President Guillermo Lasso on June 5, 2021.

**Table 4:** Power mix proposed period by period to achieve 100% renewable energy coverage by 2050.

Source	2020	2025	2030	2035	2040	2045	2050
Hydroelectric	5073.65	7000	8500	8500	8500	8500	8500
Wind	2039.7	3865	4290	5714	6159	7564	8990
Photovoltaic	275	270	3500	3500	3700	3800	4000
Biomass	80	260	1059	1369	1741	1989	2300
Tidal	2	2	2	2	2	2	2
Geothermal	477.08	522.5	663.75	854.58	1124.58	1179.16	1265
Coal	680	521.25	481.25	432.91	379.58	337.5	314.58
Natural Gas	910.08	1645.83	966.96	1015.2	1089.36	1138.32	1179.6
Oil	1768.75	2087.5	1697.5	1125.41	1038.75	392.91	0
**TOTAL (MW)**	**11306.26**	**16174.08**	**21160.46**	**22513.11**	**23734.27**	**24902.90**	**26551.18**

Article 6 of this decree specifies the priority to comply with and develop public policies and public, private initiatives, in public-private and community-type alliances that promote the energy transition towards sustainable and environmentally friendly production and consumption systems. environment, leading Ecuador towards zero net emissions by 2050. On the other hand, the legislative resolution of Escazú is approved as a Regional Agreement specifically for Public Participation, Access to Justice in Environmental Matters and Access to Information in Latin America and the Caribbean by the Republic of Ecuador.

The Escazú Agreement has the general purpose of guaranteeing the effective implementation of the rights of access to justice in environmental matters, access to environmental information and public participation in environmental decision-making processes in Latin America and the Caribbean. This is the first environmental agreement in Latin America and the Caribbean and is the first in the world to contain specific provisions in defense of human rights in environmental matters.

The purpose of the proposed transition process is to progressively increase the rates of renewable energies and, on the other hand, to reduce the contributions of fossil fuels. Thanks to the high potential for harnessing energy, especially wind, photovoltaic, biomass, it is possible to transform the Ecuadorian electricity system into a 100% renewable one, as proposed by Germany [44], China [45], Nicaragua [46], Finland [47], Japan [48], France [49], Portugal [50], Mexico [51], responsibly protecting employment sources and training workers to assume the new renewable plants. It is important to indicate that there are renewable sources that will provide low levels of generation such as geothermal and tidal, but they should not be neglected for any reason, in the same way they will contribute to the decarbonization of the electricity sector, which will be constantly growing according to the demand that is generated. It is estimated to increase at least twice the reference year for 2050, it includes 26551.18 MW in relation to the 11306.26 MW of the reference year.

### 5.5 Connection between the current law and renewable financing programs

Based on the Constitution of the Republic of Ecuador, it is contemplated:

That, article 413 of the Constitution of the Republic of Ecuador, establishes that the State must promote the energy efficiency, the development and use of practices and environmentally clean and healthy technologies, as well as renewable, diversified, low-impact energies; That, articles 74 and 75 of the Organic Law of the Service Public Electric Power -LOSPEE-establishes the objectives pursued by energy efficiency and define the principles of efficiency policy energy that should be promoted by the national government; In this general framework, in use of what is established in the Constitution and the LOSPEE in accordance with the Energy Efficiency Law in its Chapter V, Financing is established in Article 21.- Financial Mechanism for the execution of projects in the field of Energy Efficiency, it is considered that the mechanism will be attached or will be administered by the governing body in energy matters, which will prioritize the projects that must be financed, will establish the modalities of cooperation that may be adopted and will channel available resources. Under this legal framework, a financing scheme is presented below as support for decision-making.

**Fig. 15:**
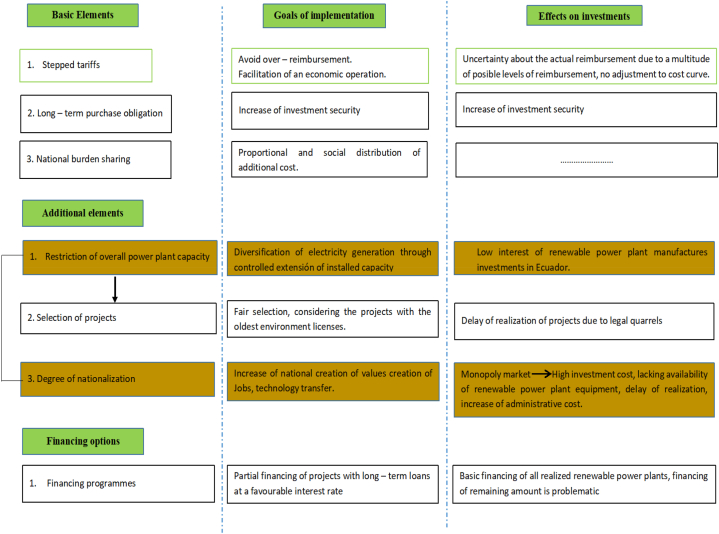
Simplified scheme for financing renewable energy plants.

The financing of long-term investments in Ecuador constitutes a great challenge under current macroeconomic conditions. As close a connection as possible between the electric power public service law and financing programs could reduce financing costs and also make the execution of renewable energy projects attractive. The granting of loans by international organizations, especially by development banks within the framework of a financing program (see Fig. 15). Revenue that becomes available from the reimbursement segment in the first years of operation it could be used in part to pay other credits in the financing framework, while the remaining amount can be used to pay interest rates. The share of new renewable energy generation plants would become more attractive and would boost the Ecuadorian economy, the cash flow would tend to improve in the first years of operation.

An official loan program in South America can generate development in investment schemes, this has been discussed for more than a decade and it is possible that it will become a reality in the medium term. However, in the first instance, other banks will have to be resorted to, possibly banks like the IDB could be a good option, it could fundamentally improve the financing framework.

## 6 Case analysis and discussion

Thanks to the new constitution of 2008, which is much more guaranteeing of rights, it has been possible to partially apply the policies and, based on the Reformed Law previously expressed and analyzed, it has solved some of the problems faced by the Ecuadorian electricity system, especially in terms of which corresponds to starting up new renewable energy generation projects. Among the projects that stand out in addition to hydroelectricity are wind energy, solar photovoltaic and biomass projects. Although future generation projects with non-conventional sources were detailed in this article as stated in the law, new generation proposals have not yet been defined in other parts of the country that could be developed in the long term. There are aspects mainly related to the financing of new generation plants that need to be implemented in view of the growing demand for energy in all sectors of the country. These and other problems facing the generation are presented below.

1) It is necessary that in Ecuador users be encouraged to also implement their generation systems and be able to contribute to the network in times of excess generation and be discounted in favor of the user.
2) At the time the renewable energy generation projects are launched in Ecuador, it is important that the sources of financing are identified, the commitments must be specified and be clearly documented in agreements, minutes, acceptance of risks between the parties, among others.

LOSPEE still maintains barriers, among these barriers real incentives are not identified as if they have the European countries. It is identified that without a regulatory framework specifically for Renewable Energies, it is very difficult to transform the Ecuadorian energy matrix therefore should be adopted mechanisms aimed at.

- Progressively reducing the share of electricity generated from oil and complete an energy transition by 2050 with 100% renewable energy.
- Secure the gradual increase in the share of solar energy photovoltaic connected to the LV network in the mode of the distributed generation, in the total contribution of electricity, of way to reduce losses, increase efficiency, improve the tension profile in the lines where the technology, save oil and reduce CO2 emissions to the atmosphere.
- Guarantee the participation of wind energy on a small and large scale, at sites identified as having potential for useable wind with these technologies.
- Give way to legal recognition of independent producers deprived of energy at the expense of the use of sources non-conventional renewables, provide guaranteed access to the grid and priority, for independent suppliers of energy using renewable sources.
- Arrange a rate special for the payment of energy to suppliers independent, which constitutes a stimulus to investment in renewable sources.
- Promote and prioritize the financing of projects focused on the study and research of the potential of renewable sources of energy, especially the geothermal, tidal and biomass.

According to the results obtained in the projection of renewable energy, it can be identified that they are fully pigeonholed in the Generation Expansion Plan based on the Policies and Strategies for the Change of the Energy Matrix of Ecuador, the Sectoral Agenda of the Electricity Sector and the Ecuadorian National Development Plan. The results are also framed in the Constitution, through which global expansion decisions can be made and the rights of nature emanated in the same Constitution of the Republic are protected. The results also allow us to glimpse the energy future of Ecuador as an energetically self-sustaining country (autarky), with projections of change in its energy matrix and with possibilities of to be an energy exporter in the region.

Based on the agreements established at the Paris Summit and ratified at COP 27, a medium-term planning has been developed, foreseen by the Ministry of Energy for the period 2018–2027, including a path to reduce the current coverage gap, which is close to 98%. However, it has been determined in this study that it is possible to start structuring programs and projects that allow it to reach 100% coverage by 2050 with 100% renewable energy. In order to meet the goals established by the United Nations Sustainable Development Goal No. 7, it is necessary to start executing projects, especially those of wind and photovoltaic solar energy to diversify the energy matrix.

## 7 Conclusions

64.21% of the total effective electrical power generated in Ecuador in 2020 corresponds to renewable energy systems. This becomes an important strategic component within the Ecuadorian electricity production system. However, analyzed source by source, the greatest contribution is hydroelectric with 5064.16 MW of effective power of the total of 5254.95 MW, which implies 96.36% of the total renewable energy. This percentage highly dependent on hydroelectricity generates an alert and it is not highly recommended to depend directly on a single source. Having analyzed the wind and solar generation potentials, it is highly recommended to take better advantage of these sources, in fact there are already experiences in Ecuador, among them the Villonaco wind power plant in Loja with 16.5 MW, Baltra in Galapagos with 2.25 MW, in San Cristobal the 2.45 MW photovoltaic project and the last one being built in Huascachaca of 50 MW. The LOSPEE clearly establishes the parameters to agree on electricity generation and the competence of the State in the electricity generation service in Ecuador.

The Ecuadorian case is a typical case of the structural contradiction that oil-exporting countries face when they are willing to start a low-carbon energy transition. On the one hand, they remain give up oil exploitation when there is a rise in oil prices and rather the ambition to exploit other oil fields arises, in many cases in protected areas, as they are already beginning to do in the eastern region. On the other hand, the discourse of promoting an energy transition is maintained, knowing that with concrete actions it is the most appropriate way, although the results are seen in the long term, reflected in the well-being of citizens, care for the environment and economic growth.

This article can be a reference for decision makers, among them: legislators, national and foreign investors, decentralized autonomous governments. This article will be a reference for other countries whose purpose is to transform their energy systems into more environmentally friendly by modifying their legislation and policies according to their realities.

It is recommended as future work, to analyze the current legislation that aspects can contribute to the residential sector becoming self-producer, in reality these aspects have generated a lot of development in European countries especially. However, in Ecuador it is not yet well defined, nor are the incentives that users can achieve by incorporating their renewable energy systems in their homes, typically solar energy is incorporated. It is important to analyze the law and its regulations for these self-generation purposes as future work.

## Author contribution statement

Daniel Icaza-Alvarez: Conceived and designed the experiments; Wrote the paper.

Francisco Jurado: Analyzed and interpreted the data.

Carlos Flores: Performed the experiments.

Geovanny Reivan Ortiz: Contributed reagents, materials, analysis tools or data.

## Data availability statement

Data included in article/supp. material/referenced in article.

## Declaration of competing interest

The authors declare that they have no known competing financial interests or personal relationships that could have appeared to influence the work reported in this paper.

## Nomenclature

**GDP**: Gross domestic product
**MW**: Megawatts
NCREB: Non-Conventional Renewable Energy Block
**MEER**: Ministerio de Electricidad y Energía Renovable (Ministry of Eectricity and Renewable Resources)
**ARCONEL**: Agencia de Regulacion y Control de Electricidad (Electricity Control and regulation agency)
MERNNR: Ministerio de Energía y Recursos Naturales no Renovables.(Ministry of Energy and Non-Renewable Natural Resources)
**CAN**: Comunidad Andina de Naciones (Andean Community)
**KWh**: kilowatt-hour
**MWh**: Megawatts-hour
CENACE: Operador Nacional de Electricidad (National Center for Energy Control)
**PME**: Plan Maestro de Electricidad 2019 (Electricity Master Plan 2019)
**MAATE**: Ministerio de Ambiente, Agua y Transición Ecológica (Ministry of Environment, Water and Ecological Transition)
**ARCERNNR**: Agencia de Regulación y Control de Energía y Recursos Naturales no Renovables (Agency for the Regulation and Control of Energy and Non-Renewable Natural Resources)
